# Novel Dicyano-Phenylenevinylene Fluorophores for Low-Doped Layers: A Highly Emissive Material for Red OLEDs

**DOI:** 10.3390/polym11111751

**Published:** 2019-10-25

**Authors:** Rosita Diana, Barbara Panunzi, Francesco Marrafino, Stefano Piotto, Ugo Caruso

**Affiliations:** 1 Department of Agriculture, University of Napoli Federico II, 80055 Portici, Italy; rosita.diana@unina.it; 2 Department of Pharmacy, University of Salerno, 84084 Fisciano, Italy; fmarrafino@unisa.it (F.M.); piotto@unisa.it (S.P.); 3 Department of Chemical Sciences, University of Napoli Federico II, 80126 Napoli, Italy; ugo.caruso@unina.it

**Keywords:** DR, OLED, fluorophore, aggregation-caused quenching (ACQ), dicyano-phenylenevinylene

## Abstract

Two efficient deep red (DR)-emitting organic dicyano-phenylenevinylene derivatives with terminal withdrawing or donor groups were synthesized. The spectroscopic properties of the neat solids and the low-doped layers in polystyrene or polyvinylcarbazole host matrixes were analyzed, and the luminescence performance was explained using density functional theory (DFT) analysis. A noteworthy 89% fluorescence quantum yield was observed for the brightest red-emissive polyvinylcarbazole (PVK) blend. This result pushed us to successfully produce an emissive red organic light-emitting device (OLED) as a preliminary feasibility test.

## 1. Introduction

Organic light-emitting devices (OLEDs) are widely regarded as next-generation technology for lighting devices [1,2] and displays [3,4], and as cutting-edge technology in bio-medicines [5,6], in security and communications [7,8], and in biological and environmental applications [9,10,11,12].

Many researchers proposed fluorescent and phosphorescent emitters for the active layers of OLEDs. Several phosphorescent transition-metal complexes, luminescent rare earth metal complexes, fluorescent organic dyes, and conjugated polymers were reported [13,14,15,16,17,18,19,20,21,22,23,24,25,26,27]. Stability, easy processability, flexibility, low operating voltages, high resolution, fast response time, wide viewing angles, color purity, luminous efficiency, and low-cost construction are the features required for emissive layers. As a result, there are still investigations into emitters able to meet all these requirements. In display technology, a mix of three colors (blue (B), green (G), and red (R)) is required to produce full-color RGB emission. Each kind of luminophore possesses unique characteristics, which must be properly mixed to obtain a white emission. Fluorescent red emitters are a limitation to realizing an efficient full-color display. Their large dipole moments produce a high degree of self-quenching, and the energy gap law decreases photoluminescence quantum yields (PLQYs) as emission red-shifts [28,29]. Organic donor–acceptor red fluorophores have a narrow band gap; hence, the formation of excimers and exciplexes dramatically reduces the luminescence performance. Pure organic materials generally possess the advantages of low cost, structural flexibility, and environmental friendliness; however, efficient red and deep red (DR) emissions are still more difficult to obtain. State-of-the-art luminescence quantum yields for DR OLEDs, with Commission Internationale de L’Eclairage (CIE) coordinates close to the National Television System Committee (NTSC)-recommended standard red CIE coordinates of (0.67, 0.33), are around 20% [30]. As a result, there is an urgent demand for red organic emitters with high luminescence efficiency and color purity.

Among the most widely utilized red emitters, organic planar polycyclic skeletons with extended π-systems or strong donor–acceptor frameworks were reported [31]. In particular, cyano-substituted π-conjugated structures were demonstrated to be able to display red emission in the solid state [32,33]. The cyano group is a strongly electron-withdrawing moiety largely employed for the design of highly polar D–A (donor–acceptor) type materials [34,35]. In previous articles [36], upon adding cyano groups to a phenylenevinylene skeleton, fluorescent emission was improved, and the wavelength emission maximum shifted in the red region. Interestingly, in symmetrical dicyano-substituted phenylenevinylene (PV) structures, the terminal substituents were found to play a relevant role in the quantitative and qualitative solid-state photoluminescence (PL) performance [37].

**Scheme 1:**
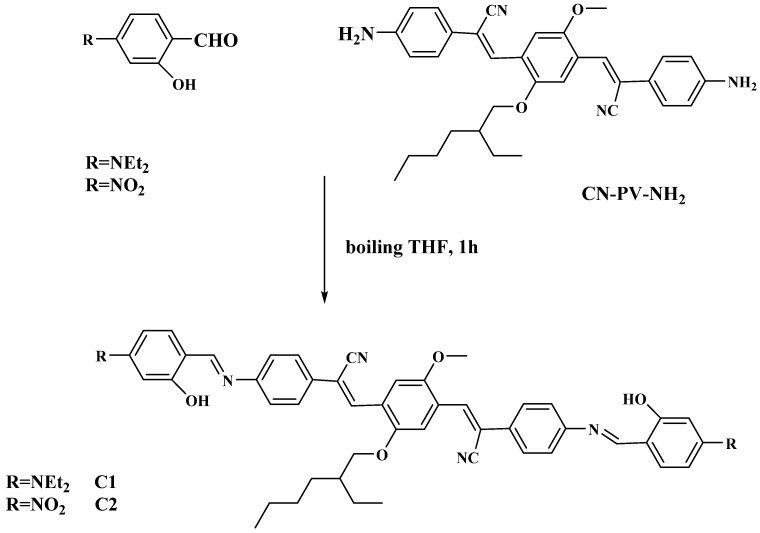
Structure of fluorophores C1 and C2.

To address red color purity and good PL performance, we synthesized two symmetrical disubstituted fluorophores, C1 and C2 in Scheme 1, based on the same dicyano-phenylenevinylene scaffold. The terminal groups were donor (N(Et)_2_) or withdrawing (NO_2_) moieties. The dicyano-phenylenevinylene skeleton showed very different PL properties depending on the substituents. An exhaustive qualitative and quantitative analysis of the emission performance was carried out in the solid phase, correlating PLQYs with computational study results.

The target of high luminescence performance was achieved by doping the dyes into host polymer materials. The problem of aggregation-caused quenching (ACQ) effect was solved. Thanks to a good choice of the dye percentage in different host matrixes (polystyrene, PS, or polyvinylcarbazole, PVK) we achieved both color purity and high PL performance. PLQYs of C1 and C2 increased by low-doping the dyes into the hole-transport polymer PVK, raising to 89% for the C1-PVK blend, a noteworthy value for a red-emitting layer, CIE (0.60, 0.37). The easily workable synthetic procedure and the high chemical and morphological stability of the polymeric blends prompted us to make a preliminary red OLED fabrication. From the C1-PVK blend, a basic device, still to be optimized, was produced. The device actually works, but does not show remarkable luminous efficiency. Nevertheless, it was used to verify the true potential of the blend as an emissive layer. Unlike other examples of very promising structures not tested in actual applications, we are able to prove that the potential of the molecule is real and not only theorical.

## 2. Results and Discussion

### 2.1. Synthesis and Optical Properties of the Fluorophores

The fluorophores C1 and C2 were obtained as summarized in Scheme 1, via condensation of the diamino derivative CN-PV-NH_2_ with 4-(diethylamino)-2-hydroxybenzaldehyde and 2-hydroxy-4-nitrobenzaldheyde, respectively. Identification and purity degree evaluation were assessed by elemental analysis, mass spectrometry, and ^1^H NMR. Phase behavior was examined by optical observation and DSC/TGA analysis. All materials were thermally stable up to 320 °C under nitrogen flow with high (above 250 °C) melting points. Compound C1 had a D–A–D–A–D skeleton, while C2 was characterized by a different A–D–A pattern.

The terminal *salen* Schiff base groups contained two sites undergoing ESIPT (excited-state intramolecular proton transfer). The fast proton transfer in ESIPT probes, inducing nonradiative deactivation, usually provides strong emission in solution but weak solid-state emission. As reported in a recent approach [38,39], it was found that sterically cluttered groups able to restrict the intramolecular rotation (RIR effect) can also force ESIPT dyes to provide solid-state emission.

Due to the bulky substituents on the PV skeleton, C1 and C2 resulted in conforming to this model. They were soluble in common organic solvents, and, in THF, they showed emission in very different regions. C1 was a DR/NIR (the NIR band peaked at 703 nm) emitter, and C2 was a yellow emitter. The absorption and emission maxima in solution are reported in Table 1. A scarce solvatochromic effect depending on the polarity of the solvents was recorded on both samples. In natural light, both solutions appeared yellow with relevant Stoke’s shifts (about 150 nm from absorption to emission absolute maxima for C1). Photoluminescence quantum yields (PLQYs) measured in THF solution using relative methods, with zinc phtalocyanine as the standard [40], were very similar (5.2% and 4.0%, respectively).

A systematic qualitative and quantitative analysis of the emission properties was carried out in the solid phase. The photophysical characterization of the neat solid compounds was performed on spin-coated thin films, obtained as described in Section 3. Both samples were red in natural light and emitted in the DR region (maxima at 651 and 638 nm, respectively). PLQYs for the neat crystalline samples were preserved with respect to the values recorded in solution (4.8% and 3.5%). Conceivably, the presence of the alkoxy chains on the central ring and the two cyano substituents suppressed close packing in the crystals (see discussion below) and guaranteed a PL response in the solid state. Nevertheless, solid-state PLQYs can be further implemented by introducing amorphous domains in the materials. Polymeric blends represent an alternative approach for use in semiconducting devices [41]. In many reports on blended organic or inorganic LEDs, the economic and synthetic advantage of fabricating high-performance polymeric blends by dissolving or micro-dispersing dyes in amorphous polymeric matrixes was demonstrated [42,43,44].

**Figure 1:**
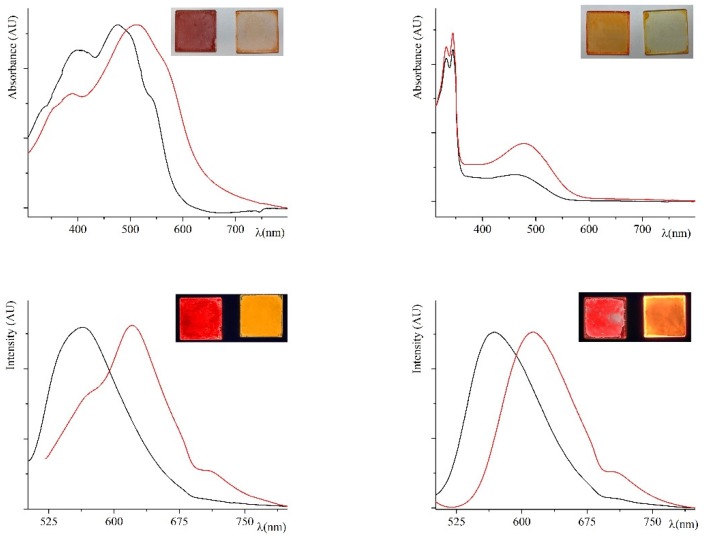
Absorption (above) and emission (below) curves of 10 wt.% blends of C1 (red curves) and C2 (black curves) in polystyrene (PS; left) and in polyvinylcarbazole (PVK; right). In the insets, photos of the correspondent C1 and C2 blends are shown.

From the crystalline samples, two kinds of doped films were prepared by spin-coating. The samples were obtained from crystals dissolved in a non-emissive (PS) and in an emissive (PVK) polymeric matrix typically employed in OLEDs. Two different percentages of fluorophores were adopted, 20% or 10% by weight (Table 2), to test the effect of the dilution in the solid state. All doped layers were homogeneous transparent films displaying various shades of yellow in natural light (Figure 1). They showed broad emission bands in the solid state, with maxima peaking in the red and orange regions for C1 and C2 blends, respectively. Interestingly, C1 blends of both matrixes were DR emitters, red-shifted by over 54 nm in the emission maxima with respect to the analogous C2 blends. Stoke’s shift values ranged from 89 to 138 nm.

Not unexpectedly [39], the 20% doped samples showed emission maxima very similar to the 10% samples. For higher concentrations PLQYs dramatically decreased. In our previous experience with analogous π-conjugated scaffolds, the aggregation-caused quenching (ACQ) effect did occur for the neat fluorophores. In this case, the dyes were weakly luminescent in the solid phase due to the strong dipole–dipole interactions, causing the formation of detrimental excimers and exciplexes. The use of fluorophores dissolved in a host polymer usually represents the right way to prevent the ACQ effect [45,46] in the solid phase, resembling a diluted solution of the fluorophores frozen in a polymeric matrix [36]. In the present case, the ACQ effect was lowered in the doped films thanks to the dilution of the fluorophores in the host matrixes, and the best results were obtained with 10 wt.% in dye. For higher concentrations, the ACQ effect prevailed. The best results for both fluorophores were obtained with the most diluted PVK blends. PLQYs measured on PS blends films were in the medium level of fluorophores and lower with respect to the analogous PVK-doped samples. In all cases, the blends of the nitro derivative showed lower PLQYs with respect to the diethylamino-based samples. This behavior is discussed in Section 2.2. The 10 wt.% C1-PVK blend guaranteed an excellent PL response in the solid state (89%), among the highest values reported in the literature for purely organic red emitters [47,48]. From an operational point of view, the use of low-doped PVK layers guarantees low cost, ease of fabrication, and high processability. According to these results, fluorophore C1 seems the perfect candidate to produce an emissive layer for a red OLED.

### 2.2. Computational Studies

Compound excitation energies were calculated with the TDDFT approach at the DFT level, using adiabatic local density approximation. Calculations were performed in THF as the simulated solvent. The main optoelectronic properties calculated for C1 and C2 are shown in Table 3.

**Figure 2:**
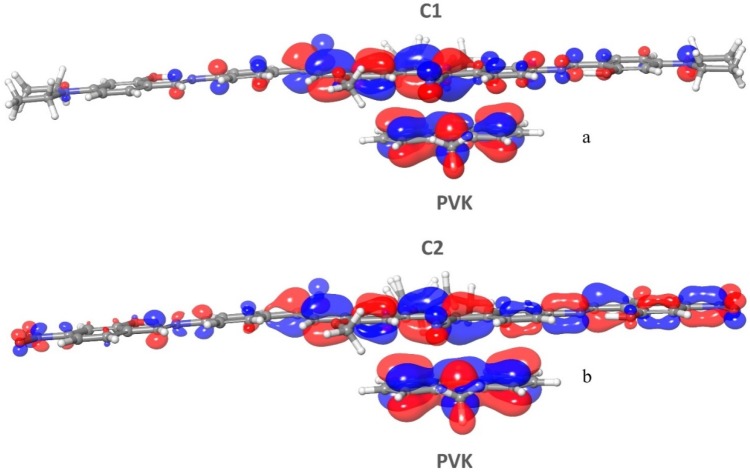
Frontier orbital representation for systems C1 (**a**) and C2 (**b**).

C1 HOMO was delocalized over the entire conjugated backbone, with a very small contribution from cyano groups. LUMO delocalization mainly covered the central rings and electron-withdrawing cyano groups. HOMO → LUMO was the main transition for the two compounds, at 568 nm for C1 and at 546 nm for C2. The C1 ultraviolet–visible light (UV–Vis) spectrum showed an additional peak at 401 nm, due to HOMO–1 → LUMO transition. C2 showed similar HOMO delocalization compared to the diethylamino derivative, with a more pronounced delocalization over the central ring oxygen atoms and minor localization over the terminal nitrogen atoms. The nitro group electron-withdrawing effect had great impact on LUMO, extending the orbital delocalization to the terminal groups compared to the diethylamino derivative (Figure 2).

The difference between C1 and C2 LUMO delocalization was clear, as shown in Figure 2. C2 showed a higher HOMO–LUMO gap and smaller HOMO and LUMO energies with a less pronounced effect on LUMO, due to the nitro group electron-withdrawing effect. This is beneficial for electron injection. Electron withdrawal in a redox reaction was observed from the HOMO. The electron-withdrawing nitro groups were responsible for the higher oxidation potential observed for C2. The two compounds showed typical hole and electron reorganization energies (HRE and ERE, respectively) for the class of compounds. However, nitro groups caused a decrease in the ERE. The hopping rate is governed by various parameters, one of which is the reorganization energy (RE). HRE and ERE are strictly related to the anion and cation state geometries, and the RE is defined as the energy difference between the charged and neutral systems at the two different geometries.

A small RE means that the compounds have high carrier mobility. The energies are proportional to the geometry deformation during the charge transfer process. In C2, the ERE was smaller than the HRE. This means a smaller geometrical deformation upon hole injection compared to electron injection, while C1 showed the opposite behavior. Furthermore, C1 had a smaller hole extraction potential (see Table 3), indicating that hole injection into C1 was slightly easier than in the nitro derivative. Also, a slightly higher maximum electron extraction potential made electron injection easier in C1. The results indicate that the electron-withdrawing nitro groups altered the system electron-transporting properties.

Absorption and emission maxima were very similar for the two derivatives. Emission values were in good agreement with experimental values, while absorption values were quite higher than experimental data, which could possibly be addressed by selecting a targeted basis set.

**Figure 3:**
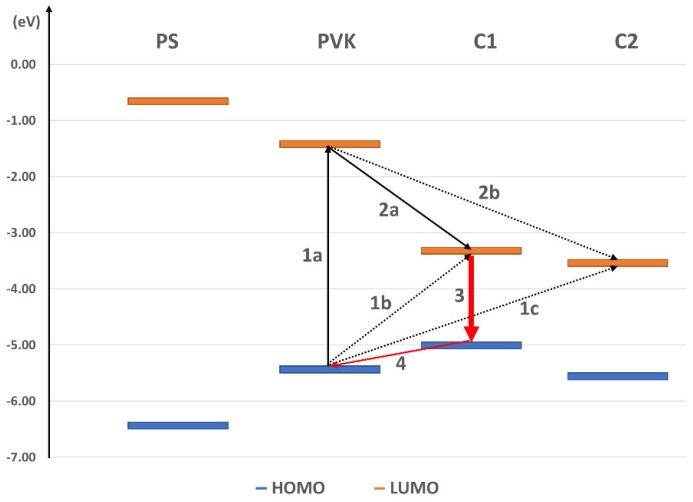
Energies of orbital levels of PS, PVK, C1, and C2. The arrows indicate possible electronic transitions.

The energy levels of the molecular orbitals of C1 and C2 and their spatial locations may justify the high quantum yield of C1-PVK blends. Unlike in PS, in PVK blends, a strong interaction with the carbazole ring of the polymer was possible. The distance between PVK and C1 or C2 was around 3.4–3.5 Å. Figure 2a,b represent the boundary orbitals of the carbazole pair in C1-PVK and C2-PVK systems, respectively. The energy levels of the HOMO and LUMO of all the molecules considered are shown in Figure 3. The PS matrix was not able to interact with the dispersed molecules, unlike the PVK matrix. The most relevant absorption was the HOMO–LUMO absorption of PVK (transition 1, Figure 3). The system could relax into the LUMO of C1 and C2 (transitions 2a and 2b). The subsequent radiative emission (transition 3) was only possible if electrons were removed from the LUMO of C1 and C2. This was possible for C1 (transition 4), because its HOMO had higher energy than the HOMO of the PVK, but not for C2, which had a HOMO energy lower than the corresponding HOMO of the PVK.

Interestingly, this analysis could offer a rational of the blend system PL performance, and allows a prediction on the optimal matrix for the system, once the orbital levels of the dopant are defined. It is important to note that unusually high PLQYs were previously observed in red emitters [49]. In that case, the high PLQY was explained by a reduction of the activation barrier on the deactivation path to the conical point of intersection, in accordance with the Bell–Evans–Polanyi. This was not the case with our C1-PVK system. In fact, C1 (as well as C2) did not show an interesting quantum yield for the isolated molecule (see Table 1), but did so only in solution with PVK (Table 2). The presence of spatially close carbazole rings and PVK HOMO energy levels slightly lower than C1 were the requirements for the observation of a high quantum yield.

### 2.3. Fabrication of a Red OLED

**Figure 4:**
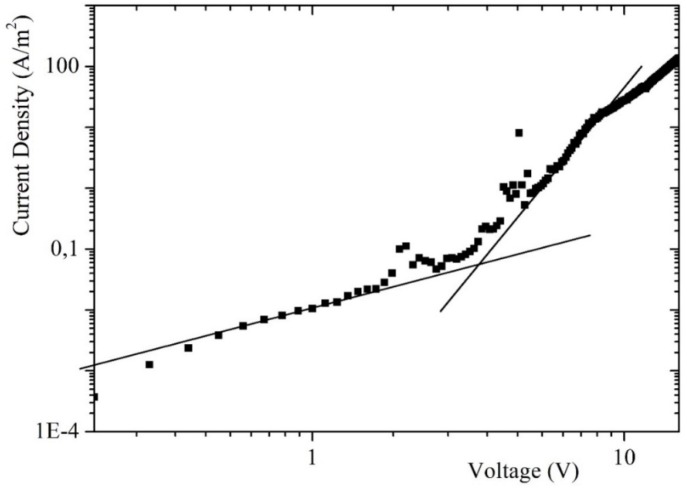
Current density versus voltage characteristic of the device realized with the 10 wt.% C1-PVK blend.

An OLED device with the simplest architecture was fabricated with the 10 wt.% C1-PVK blend as an emissive layer. The structure of the device is reported in Section 3.3. In Figure 4, the current density–voltage characteristic of the device is reported. The turn-on voltage was approximately 3.5 V. The current density magnitude was quite low with respect to analogous devices reported in the literature [50], probably because the thickness of the layers was not optimized in the device structure. Moreover, since the measurements were performed in air soon after the evaporation, the degradation of the Ca/Al cathode could have affected the series resistance of the whole device, further limiting the current density.

**Figure 5:**
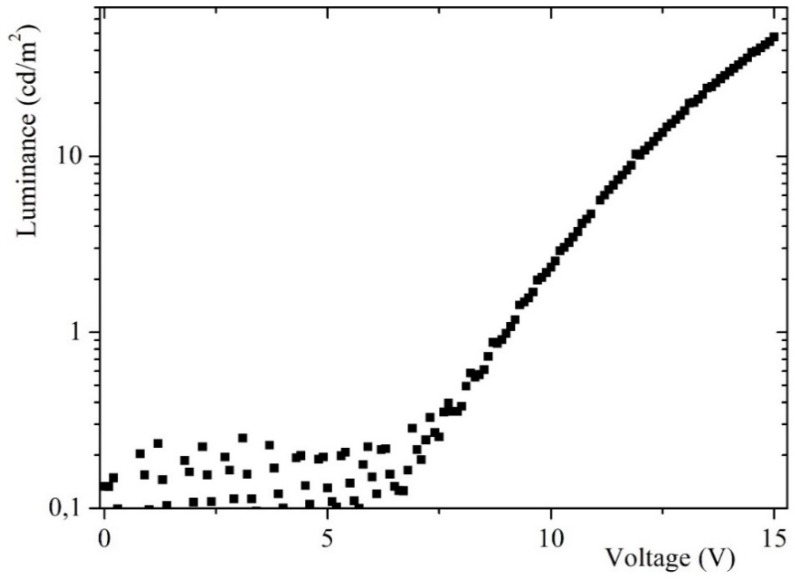
Luminance versus voltage characteristic of the device realized with the 10 wt.% C1-PVK blend.

**Figure 6:**
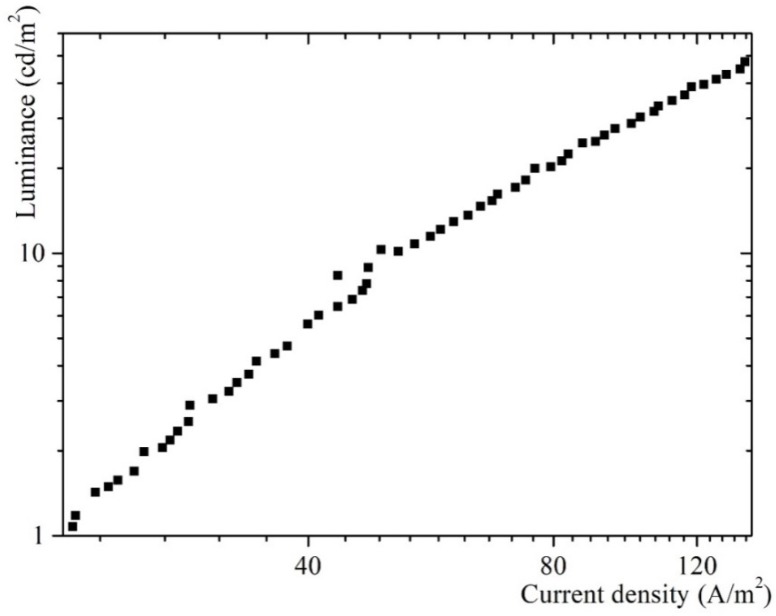
Luminance versus current density characteristic of the device realized with the 10 wt.% C1-PVK blend.

**Figure 7:**
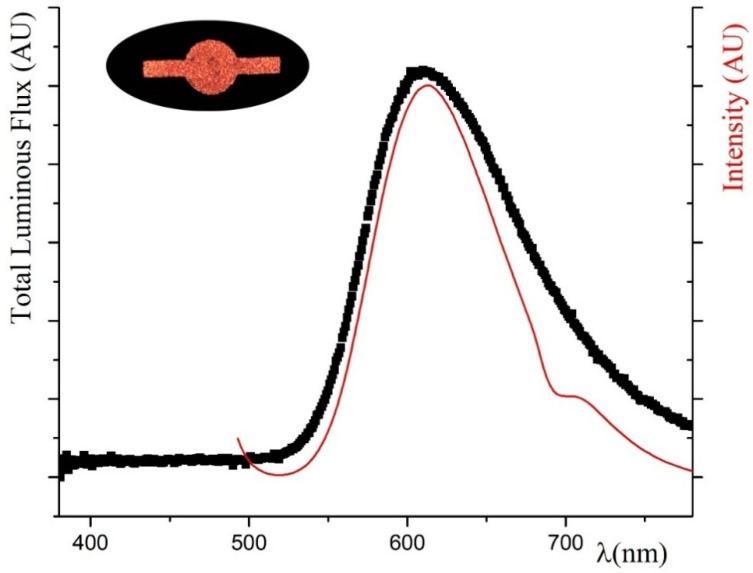
Electroluminescence intensity (normalized) versus wavelength characteristic of the device realized with the 10 wt.% C1-PVK blend. The photoluminescence (PL) spectrum of the same sample is shown in red. The red organic light-emitting device (OLED) is shown in the inset.

In Figure 5, the luminance versus voltage characteristic of the device is reported. The optical turn-on voltage was around 7 V. Also, the absolute values of the luminance were affected by the limitation of the current density. Interestingly, the current efficiency (calculated as the slope of the line in Figure 6) was a value that can be considered as a state-of-the-art PVK-doped layer (0.44 cd/A) [50]. In fact, the current efficiency was only slightly decreased with respect to a pristine PVK active layer due to the addition of C1 dye, probably because of our good PLQY, especially considering the case of doped emissive layers [50]. In Figure 7, the normalized electroluminescence intensity versus wavelength is reported, as well as the corresponding very similar PL spectrum of the C1-PVK blend. This spectrum indicates that the electroluminescence emission was mainly due to the red fluorophore. The PVK matrix promoted exciton formation only in the dye sites, considering that PVK fluorescence peaked at 400 nm.

Despite being a basic OLED, the device worked with a good current efficiency, and its performance can be improved once the manufacturing parameters (such as layer thickness, implementation of electro-blocking and hole-blocking layers, encapsulation, etc.) are optimized.

## 3. Materials and Methods

All starting products were commercially available. Firstly, 2-hydroxy-4-nitrobenzaldehyde was obtained as described [36,51]. ^1^H NMR spectra were recorded in DMSO-d_6_, with a Bruker Avance II 400 MHz apparatus (Bruker Corporation, Billerica, MA, USA). Mass spectrometry measurements were performed using a Q-TOF premier instrument (Waters, Milford, MA, USA) equipped with an electrospray ion source and a hybrid quadrupole time-of-flight analyzer. For optical observations, a Zeiss Axioscop polarizing microscope (Carl Zeiss, Oberkochen, Germany) was employed. A DSC/TGA Perkin Elmer TGA 4000 (PerkinElmer, Inc., Waltham, MA, USA) with a scanning rate of 10 °C/min provided phase transition temperatures and enthalpies, as well as the decomposition temperature (*T*_d_) measured under nitrogen flow, assumed as the temperature where the 5 wt.% weight loss was recorded. UV–Vis and fluorescence spectra were obtained using JASCO F-530 and FP-750 spectrometers (JASCO Inc., Mary’s Court, Easton, MD, USA). The crystalline powders of dissolved (C1 or C2) *o*-dichlorobenzene and PS or PVK were mixed at 10 or 20 wt.% in dye. Thin films of the neat samples and of the blends were obtained via the spin-coating technique using an SCS P6700 spin-coater (Specialty Coating Systems Inc., Indianapolis, IN 46278, USA) operating at 600 RPM for one minute and 1200 RPM for one minute, before annealing for 10 min at 120 °C. PS (molecular weight 18,700 Da) and PVK (molecular weight 1100 Da) were commercially available, supplied by Sigma Aldrich (Sigma-Aldrich Corporation, St. Louis, MO, USA).

### 3.1. Synthesis of C1 and C2

The same general procedure was employed for C1 and C2. C1 is described as an example. To 3.76 g (0.01 mmol) of CN-PV-NH_2_, suspended in 30 mL of dry THF, 3.87 g (0.02 mmol) of 4-(diethylamino)-2-hydroxybenzaldehyde was added under stirring at room temperature. After 1 h, the crude product precipitated at room temperature. The compound was crystallized by dichloromethane/hexane and was further purified by washing with ethanol. T_m_ = 255 °C; T_d_ = 360 °C. ^1^H NMR (400 MHz, DMSO-d_6_, 25 °C, ppm): 1.35 (t, 3H), 1.37 (t, 3H), 1.46 (m, 18 H), 1.67 (m, 2H), 2.02 (m, 1H), 3.94 (m, 8H), 4.47 (s, 3H), 4.59 (m, 2H), 6.52 (s, 2H), 6.84 (d, 2H), 7.86 (d, 2H), 7.90 (d, 4H), 8.24 (t, 4H), 8.42 (s, 2H), 8.53 (d, 2H), 9.18 (s, 2H), 9.98 (s, 2H). HRMS (ESI): *m*/*z* calculated for C_55_H_62_N_6_O_4_ + H^+^: 871.48; found 871.36 [M + H]^+^. Elemental analysis calculated (%) for C_55_H_62_N_6_O_4_: C, 75.83; H, 7.17; N, 9.65; found C, 75.70; H, 7.05; N, 9.63.

C2: T_m_ = 314 °C; T_d_ = 320 °C. ^1^H NMR (400 MHz, DMSO-d_6_, 25 °C, ppm): 1.34 (t, 3H), 1.38 (t, 3H), 1.70 (m, 6 H), 1.75 (m, 3H), 4.45 (m, 5H), 7.00 (m, 2H), 7.80 (m, 2H), 7.90 (d, 4H), 8.23 (m, 4H), 8.45 (s, 4H), 8.53 (d, 2H), 9.20 (s, 2H), 9.68 (s, 2H). HRMS (ESI): *m*/*z* calculated for C_47_H_42_N_6_O_8_ + H^+^: 819.31; found 819.20 [M + H]^+^. Elemental analysis calculated (%) for C_47_H_42_N_6_O_8_: C, 68.94; H, 5.17; N, 10.26; found C, 68.74; H, 5.05; N, 10.43.

### 3.2. Computational Studies

Quantum-mechanical calculations were performed using the Jaguar package, Schrödinger Release 2017-4 [52], at the DFT/B3LYP theory level, optimizing molecular geometry with the B3LYP functional and LACVP** basis set. Charges were assigned through the NBO approach. Single-point calculations were executed on optimized geometries using Dunning’s correlation-consistent triple-ζ basis set cc-pVTZ (-f), which includes a double set of polarization functions. Absorption values were obtained from vertical excitation energy values, calculated at neutral compound geometry with TDDFT and Tamm–Dancoff [53] approximation. A Poisson–Boltzmann solver (PBF) [54] was used to simulate the solvent THF. Computed redox data were used to calculate “scaled” HOMO and LUMO energies, through the following equations:

Absolute electrode = electrode potential + NHE energy,

Orbital energy = redox potential + absolute electrode,

where “NHE energy” represents the energy of the NHE electrode in water (−4.28 V), and “electrode potential” represents the potential of choice electrode relative to NHE.

### 3.3. OLED Fabrication

The structure of the device was glass/ITO/ poly(3,4-ethylenedioxythiophene):poly(styrenesulfonate) (PEDOT:PSS)/PVK:red/Ca/Al. The ITO-coated Cornings Eagle XG glass substrates, purchased from Delta Technologies, LTD, with a sheet resistance lower than 10 Ω/square, were firstly cleaned with detergent, ultrasonicated in acetone and isopropanol, and subsequently dried in an oven. After a selective etching of a part of the ITO to define the front electrode, films of poly(3,4-ethylenedioxythiophene):poly(styrenesulfonate) (PEDOT:PSS), purchased from Heraeus Clevios™ P VP AI 4083, were spin-coated at 5000 rpm, after passing through a 0.45-μm filter. The thickness of the PEDOT:PSS layer was 40 nm. The samples were dried for 10 min at 150 °C in air.

The emissive layer (PVK:red; 20 mg/mL, in 1,2-dichlorobenzene) was deposited by spin-coating. For PVK:red, a 140-nm-thick layer was obtained. The Ca electrode (20 nm) capped with Al (100 nm) was deposited on the active layer by thermal evaporation in ultra-high vacuum (10^−7^ mbar) using a shadow mask to form a top anode. The energy levels of PEDOT:PSS and Ca were respectively −5.2 eV and 3.35 eV. The active area of the device, defined as the superposition of the two electrodes, was 22 mm^2^.

Current–voltage (I–V) and electroluminescence–voltage (EL–V) characteristics were measured using a Keithley 2400 power supply source meter with constant increment steps (0.1 V). Electroluminescence analysis was performed using a photodiode (Newport810UV) connected to a Keithley 6517A Electrometer. The device electroluminescence spectra were evaluated through an integrated sphere connected to a spectroradiometer (Optronic Laboratories Inc., Orlando, FL 32811, USA). The film thicknesses were measured using a KLA Tencor P-10 surface profiler (KLA Corporation, Milpitas, California 95035, USA). The experiments were conducted in air on the unencapsulated device.

## 4. Conclusions

By combining a dicyano-phenylenevinylene scaffold with terminal withdrawing or donor groups, we successfully developed two efficient yellow and red organic emitters, employable as low-percentage dopants in PS and PVK matrixes. The incorporation of the fluorophores into host polymers increased the PLQYs up to a noteworthy value of 89% for the diethylamino-substituted dye in the 10 wt.% PVK blend. The DFT analysis made it possible to clarify the excellent PLQY of the C1-PVK blend. This material retains the desired red emission of the neat sample with CIE coordinates (0.60, 0.37). Consequently, the fabrication of a very simple solution-processed red OLED based on this blend was attempted. Despite not being technologically optimized, an example of a device with red emission and an EL spectrum which overlapped with the PL spectrum was obtained. 

## Figures and Tables

**Table 1:** Optical data for C1 and C2 recorded in solution and as crystalline neat compounds. PLQY—photoluminescence quantum yield.

Compound	λ_abs_ (nm) ^a^	λ_em_ (nm) ^b^	PLQY% ^c^	λ_abs_ (nm) ^d^	λ_em_ (nm) ^e^	PLQY% ^f^
C1	(364) 470	628 (703)	5.2 ± 0.5	382 (522)	651	4.8 ± 0.2
C2	398 (450)	563	4.0 ± 0.5	(412) 499	638	3.5 ± 0.2

^a^ Wavelength of ultraviolet–visible light (UV–Vis) absorbance maxima in THF solution. ^b^ Wavelength of emission maxima in THF solution. ^c^ PLQYs in THF solution. ^d^ Wavelength of UV–Vis absorbance maxima as a neat solid. ^e^ Wavelength of emission maxima as a neat solid. ^f^ PLQYs as a neat solid.

**Table 2:** Optical data for C1 and C2 neat solids and in polystyrene (PS) and polyvinylcarbazole (PVK) blends.

Compound		λ_abs_ (nm) ^a^	λ_em_ (nm) ^b^	PLQY% ^c^	λ_abs_ (nm) ^d^	λ_em_ (nm) ^e^	PLQY% ^f^
C1	20%	510	620	20 ± 1	478	616	78 ± 2
	10%	512	621	21 ± 1	477	615	89 ± 2
C2	20%	480	566	14 ± 1	462	570	19 ± 2
	10%	476	565	15 ± 1	459	569	25 ± 1

^a^ Wavelength of UV–Vis absorbance maxima in PS blend. ^b^ Wavelength of emission maxima in PS blend. ^c^ PLQYs in PS blend. ^d^ Wavelength of UV–Vis absorbance maxima in PVK blend. ^e^ Wavelength of emission maxima in PVK blend. ^f^ PLQYs in PVK blend.

**Table 3:** Electro-optical properties calculated on C1 and C2.

Properties	C1	C2
Oxidation potential (eV)	0.47	1.02
Reduction potential (eV)	−1.18	−1.02
Hole reorganization energy (eV)	0.24	0.26
Electron reorganization energy (eV)	0.39	0.13
Triplet energy (eV)	1.25	1.35
λAbs_max_ (nm)	568	546
λE_max_ (nm)	661	636
Scaled HOMO (eV)	−5.01	−5.57
Scaled LUMO (eV)	−3.35	−3.52
HOMO–LUMO (eV)	1.66	2.05
Triplet stabilization energy (eV)	0.43	0.33
Hole extraction potential (eV)	5.75	6.70
Triplet reorganization energy (eV)	−2.17	−2.73
Electron extraction potential (eV)	0.69	0.66
